# Is Computed Tomography Necessary Before Septoplasty? Correlation With Physical Examination and Patient Complaints

**DOI:** 10.7759/cureus.38558

**Published:** 2023-05-04

**Authors:** Mafalda Martins de Sousa, João Rebelo, Sónia Martins, Helena Silveira, Tiago Órfão, Carla Pinto Moura

**Affiliations:** 1 Otorhinolaryngology, Centro Hospitalar Universitário São João, Porto, PRT; 2 Radiology, Centro Hospitalar Universitário São João, Porto, PRT; 3 Otolaryngology, Centro Hospitalar Universitário São João, Porto, PRT; 4 Otolarhinoryngology, Centro Hospitalar Universitário São João, Porto, PRT

**Keywords:** nasal septum deviation, nose surgery, physical examination, computed tomography, nasal obstruction

## Abstract

Introduction: Septoplasty is one of the most common surgeries performed by otorhinolaryngologists. The gold standard for the evaluation of septal deviation is anterior rhinoscopy and nasal endoscopy. Frequently, computed tomography (CT) is also performed, although the correlation between septal deviation on CT and physical examination is unclear. Objectives: To study the relationship between symptoms and physical and radiological evaluation in patients who underwent septoplasty. Methods: A prospective study of patients with nasal obstruction and septal deviation who underwent septoplasty. Anterior rhinoscopy and nasal endoscopy were performed by the surgeon, and the CT was evaluated by a radiologist. The degree of obstruction was evaluated in three distinct septal locations. The Nasal Obstruction Symptom Evaluation (NOSE) score was used before the surgery and two months after the surgery. Results: The study included 43 patients, of whom 60.5% were male, with an average age of 37.09 years (±12.56). The degree of septal deviation in the physical examination was significantly different from that observed in CT (p˂0.05). Cartilaginous or maxillary crest septal deviations >75% were more commonly recognized by physical examination, while osseous septum deviations of 25%-50% were more easily detected by CT. There was no difference between the degree of septal deviation and the preoperative NOSE. The median preoperative NOSE was 60, and the postoperative was 5, with significant improvement (p<0.05).

Conclusion: CT doesn’t appear to be useful in the evaluation of septal deviation since it is different from the findings of a physical examination and isn’t associated with the NOSE score. Clinical decisions should be based on a physical examination and patient complaints.

## Introduction

Septoplasty is one of the most common surgeries performed by otorhinolaryngologists. The primary indication for septoplasty is nasal septal deviation causing nasal obstruction [1]. Since many patients have septal deviation without symptoms and others have symptoms related to another medical etiology, such as rhinitis, the clinical decision to perform a septoplasty should be based on medical history and objective evidence of septal deviation [1,2]. 

The gold standard for the evaluation of septal deviation is anterior rhinoscopy and nasal endoscopy [1,3]. In many cases, preoperative computed tomography (CT) is also performed, even though the correlation between septal deviation characteristics on the CT scan and physical examination remains unclear. CT remains the gold standard for the evaluation of paranasal anatomy and sinus pathologies [4-6]. However, the effects of radiation and increased costs have raised the question of whether routine preoperative CT is truly necessary [4]. The purpose of this work is to study the relationship between symptoms, physical examination, and radiological evaluation in patients proposed for septoplasty.

## Materials and methods

Study design

We performed a prospective study at a Tertiary University Hospital. The study protocol was approved by the Ethics Committee (324/18) and carried out in accordance with the principles of the Declaration of Helsinki.

Patient selection and evaluation

We selected consecutive patients with complaints of nasal obstruction and septal deviation who underwent septoplasty. Patients under 18 years old with chronic rhinosinusitis, rhinitis, or previous septoplasty were excluded. All surgeries were performed by the same surgeon. Anterior rhinoscopy and nasal endoscopy were performed by the surgeon in the preoperative consultation. All the CT scans were evaluated by the same radiologist, who was blinded for the physical examination.

Three distinct septal locations (cartilaginous, maxillary crest, and osseous septum) were evaluated on a 4-point scale representing (1) 0% to 25%, (2) >25% to 50%, (3) >50% to 75%, and (4) >75% obstruction. The Nasal Obstruction Symptom Evaluation (NOSE) score was applied before surgery and two months after surgery [7].

Statistical analysis

Statistical analysis was performed with the IBM Corp. Released 2019. IBM SPSS Statistics for Windows, Version 26.0. Armonk, NY: IBM Corp. Normality was evaluated by the Kolmogorov-Smirnov test, and variances by the Levene test. A descriptive analysis of the patient's characteristics was performed, taking into consideration absolute and relative frequencies for categorical variables mean and standard deviation for continuous variables. Comparison between groups was performed using the Qui-square and Cohen’s kappa coefficients to measure inter-rater reliability. A p-value of less than 0.05 was considered statistically significant.

## Results

We included 43 patients, of whom 60.5% were male, with an average age of 37.09 years (±12.56) (Table 1).

**Table 1:** Descriptive analysis of the population

Number of patients	43
Gender (number (%))	
Female	17 (39.5)
Male	26 (60.5)
Age (years ± standard deviation)	37.09 ± 12.56

The degree of septal deviation (cartilaginous, maxillary crest, and osseous) was classified as <25%, 25%-50%, 50%-75%, or >75% before the surgery. The classification of the septal deviation in the physical examination was significantly different from that observed in CT (p˂0.006). There was a significant difference between the maxillary crest deviation on physical examination and in CT (Cohen’s Kappa test, p˂0.001). Osseous septum deviation was also different on physical examination and CT (Cohen’s Kappa test, p=0.006), and there was also a significant difference between cartilaginous deviation on physical examination and in the CT (Cohen’s Kappa test, p˂0.001).

Cartilaginous or maxillary crest septal deviations >75% were more commonly recognized by physical examination, while osseous septum deviations of 25%-50% were more easily detected by CT (Qui-square, p<0.05). The median preoperative NOSE was 60, and the median postoperative NOSE was 5 (Tables 2, 3).

**Table 2:** Preoperative NOSE score

		Frequency	%
Score	No obstruction	1	2.3
	Moderate	14	32.6
	Severe	24	55.8
	Extreme	4	9.3
	Total	43	100

**Table 3:** Postoperative NOSE score

		Frequency	%
Score	No obstruction	15	34.9
	Mild	21	48.8
	Moderate	4	93
	Severe	3	7
	Total	43	100

Pre- and postoperative NOSE were significantly different (Spearman test: 0.365, p=0.016), with an improvement in the NOSE classification after surgery (Qui-square, p<0.05). There was no correlation between the severity of septal deviation (on physical examination and in CT) and the preoperative NOSE classification (Qui square, p=0.882). 

## Discussion

In patients with complaints of nasal obstruction, the nasal septal deviation is the most common pathology, and consequently, septoplasty is one of the most commonly performed surgical procedures in otorhinolaryngology [4]. The nose and paranasal sinuses have complex anatomic structures. Even though anterior rhinoscopy and nasal endoscopy are the gold standards for evaluating the nasal septum, endoscopic imaging is not available in all centers or isn’t implemented routinely for every patient [7]. Computed tomography is frequently used before septoplasty, but some surgeons have questions regarding its use in patients who don’t have symptoms of rhinosinusitis [1]. In our study, there was no significant correlation between the degree of septal deviation on physical examination and on CT. Cartilaginous or maxillary crest septal deviations >75% were more commonly recognized by physical examination, while osseous septum deviations of 25%-50% were more easily detected by CT. A possible explanation for cartilaginous and maxillary crest deviations being more easily detected in the physical examination may be the fact that CT may not have sufficient sensibility since the cuts are around 0.5 mm. Regarding the posterior osseous deviation, CT has greater acuity since it is more difficult to observe posterior deviations on physical examination. Besides that, since the collapse of the nasal valve is dynamic, it has to be observed during physical examination, and it is difficult to evaluate in CT, so we chose not to include this area. Patients’ perception of nasal obstruction is complex, so we used the NOSE score to evaluate and compare patients’ symptoms before and after surgery [8]. As should be expected, when the indication for surgery is appropriate, there is a significant improvement in the NOSE score after surgery. Our results also demonstrated that there was no correlation between the degree of septal deviation (on physical examination and CT) and the patient’s complaints, measured by the NOSE score. This finding shouldn’t surprise most otorhinolaryngologists since we frequently see patients with small deviations and notorious complaints or patients with moderate to severe deviations and little/no complaints. However, in the literature, there are conflicting results regarding this topic [8-10]. So, according to our study, it appears that CT shows no additional benefit before septoplasty in patients with nasal obstruction since there is no correlation between physical and imagiological evaluation. As expected, this doesn’t apply to patients who have chronic rhinosinusitis, for whom CT helps guide the extent of surgery that needs to be performed. This study benefits from the prospective analysis and the blinded and independent assessments of each of these patients by an otolaryngologist and a radiologist. The fact that there was only one otorhinolaryngologist and one radiologist reduces interobserver variability, which may be an advantage but also a limitation. However, the evaluation was similar to that performed in real-life conditions. Another advantage of our study is the fact that patients with symptoms or a diagnosis of chronic rhinosinusitis and those who needed further surgery from septoplasty weren’t included because we wanted to study merely the patients with nasal obstruction related to septal deviation. 

In the current literature, only a few studies have analyzed the relationship between CT and subjective perception of nasal obstruction. These studies expressed opposite attitudes about the need for CT for the evaluation of noncomplicated nasal septal deviation, with some advocating CT before septoplasty [11-13], while others didn’t [3,14,15]. To the best of our knowledge, there is only one other study to correlate not only the physical examination with CT but also the patients’ complaints [7]. This study demonstrated that neither physical examination nor CT grading of septum deviation could objectively confirm nasal obstruction severity. CT is associated with radiation exposure and an increased cost. From our data, we suggest that a physical examination associated with a thorough anamnesis and NOSE scale grading can identify patients that benefit from septoplasty without the need for performing preoperative CT.

## Conclusions

To the best of our knowledge, there is only another study to analyze the NOSE score, physical examination, and CT findings in the same cohort of patients. CT can’t substitute physical examination, the gold standard, in the assessment of septal deviation. CT doesn’t appear to be useful in the evaluation of septal deviation since it is different from the findings of anterior rhinoscopy and nasal endoscopy and has no association with the NOSE score. Clinical decisions should be based on a physical examination and patient complaints.
